# Effects of Caffeine Treatment on Hepatopulmonary Syndrome in Biliary Cirrhotic Rats

**DOI:** 10.3390/ijms20071566

**Published:** 2019-03-28

**Authors:** Ching-Chih Chang, Chiao-Lin Chuang, Ming-Hung Tsai, I.-Fang Hsin, Shao-Jung Hsu, Hui-Chun Huang, Fa-Yauh Lee, Shou-Dong Lee

**Affiliations:** 1 Division of General Medicine, Department of Medicine, Taipei Veterans General Hospital, Taipei 112, Taiwan; ccchang7@vghtpe.gov.tw (C.-C.C.); clchuang@vghtpe.gov.tw (C.-L.C.); 2 Faculty of Medicine, National Yang-Ming University School of Medicine, Taipei 112, Taiwan; yfhsin@vghtpe.gov.tw (I.-F.H.); sjhsu@vghtpe.gov.tw (S.-J.H.); sdlee@vghtpe.gov.tw (S.-D.L.); 3 Division of Gastroenterology and Hepatology, Department of Medicine, Taipei Veterans General Hospital, Taipei 112, Taiwan; 4 Chang Gung University College of Medicine and Division of Gastroenterology and Hepatology, Chang Gung Memorial Hospital, Taoyuan 333, Taiwan; mhtsai@adm.cgmh.org.tw; 5 Division of Gastroenterology, Department of Medicine, Cheng Hsin General Hospital, Taipei 112, Taiwan

**Keywords:** caffeine, hepatopulmonary syndrome, liver cirrhosis

## Abstract

Hepatopulmonary syndrome (HPS) is a lethal complication of cirrhosis characterized by hypoxia and overt intrapulmonary shunting. In this study, we investigated the effect of caffeine in rats with common bile duct ligation (CBDL)-induced liver cirrhosis and HPS. CBDL rats were randomly allocated to receive caffeine or vehicle for 14 days. On the 28th day after CBDL, mortality rate, hemodynamics, liver, and renal biochemistry parameters and arterial blood gas analysis were evaluated. Lung and liver were dissected for the evaluation of inflammation, angiogenesis and protein expressions. In another series with parallel groups, the intrapulmonary shunting was determined. Caffeine significantly reduced portal pressure (caffeine vs. control: 10.0 ± 3.7 vs. 17.0 ± 8.1 mmHg, *p* < 0.05) in CBDL rats. The mortality rate, mean arterial pressure, biochemistry data and hypoxia were similar between caffeine-treated and control groups. Caffeine alleviated liver fibrosis and intrahepatic angiogenesis but intrapulmonary inflammation and angiogenesis were not ameliorated. The hepatic VEGF/Rho-A protein expressions were down-regulated but the pulmonary inflammation- and angiogenesis-related protein expressions were not significantly altered by caffeine. Caffeine did not reduce the intrapulmonary shunting, either. Caffeine has been shown to significantly improve liver fibrosis, intrahepatic angiogenesis and portal hypertension in cirrhotic rats, however, it does not ameliorate HPS.

## 1. Introduction

Coffee, a worldwide consumed beverage, has caught attention for its potential benefits in liver diseases [1]. Increased coffee consumption is associated with the reduction of liver fibrosis in patients with chronic liver disease and the incidence of hepatocellular carcinoma [2,3]. Caffeine (3,7-dihydro-1,3,7-trimethyl-1H-purine-2,6-dione) is the major compound in coffee and many energy drinks. The anti-angiogenesis effect of caffeine has been noticed [4]. In addition, the anti-inflammatory effects of caffeine have been reported in rats challenged with lipopolysaccharide [5]. In our previous study, we found that caffeine reduced portal pressure and ameliorated portal-systemic shunting, mesenteric angiogenesis, and liver fibrosis in cirrhotic rats [6]. 

Hepatopulmonary syndrome (HPS) is characterized by hypoxia in patients with chronic liver diseases, especially in those with cirrhosis [7]. Emerging data show that the prevalence of HPS ranges from 5 to 32% in cirrhotic patients who need liver transplantation [8]. The prognosis of HPS is ominous, evidenced by a median survival of 24 months and a five-year survival rate of 23% in cirrhotic patients developing HPS [9]. HPS is characterized by overt intrapulmonary shunting due to vasodilatation and abnormal angiogenesis [10,11]. Intrapulmonary shunting is the main condition causing arterial hypoxia in HPS [12]: Unoxygenated blood can escape through the pulmonary shunting directly into the systemic circulation without gas exchange. The severity of arterial hypoxemia is related to the extent of ventilation-perfusion mismatch, intrapulmonary shunting and diffusion impairment [13]. Abnormal pulmonary angiogenesis plays a crucial role in the development and maintenance of HPS. Zhang et al. reported that pathological pulmonary angiogenesis and enhancement of vascular endothelial growth factor (VEGF) production by intrapulmonary monocytes were associated with the development of HPS in experimental animals [10]. Our previous study also showed that sorafenib alleviated experimental HPS by attenuating intrapulmonary angiogenesis through down-regulating the VEGF/VEGF-receptor 2 pathway [14]. In addition to abnormal angiogenesis, the initiation of HPS comes from overwhelming intrapulmonary inflammation. The chemokine fractalkine (CX_3_CL_1_) expression and signaling are increased in HPS rats, which contributes to pulmonary intravascular monocyte accumulation, angiogenesis and the development of experimental HPS [15]. Moreover, Thenappan et al. found that experimental HPS resulted from the intrapulmonary accumulation of CD68-positive macrophages, and the depletion of macrophages might exert a therapeutic potential [16]. Consistent with those findings, we previously reported that rosuvastatin alleviated experimental HPS through the inhibition of pulmonary inflammatory angiogenesis, which was related to the down-regulation of the tumor necrosis factor-α (TNF-α)/nuclear factor kappa B (NFκB) and VEGF/Rho-A pathways [17]. 

The common bile duct ligation (CBDL) murine model is an established animal model of liver cirrhosis and HPS [10,14,15,16,17]. Targeting the mechanism of HPS involving abnormal intrapulmonary angiogenesis and inflammation, in this study, we aimed to investigate the effects of caffeine on HPS in rats with CBDL-induced liver cirrhosis and the underlying mechanisms. 

## 2. Results

### 2.1. Mortality Rates of Caffeine- and Vehicle-Treated CBDL Rats

There was no significant difference in mortality rates between caffeine- and vehicle-treated (control) CBDL rats (control vs. caffeine: 25% (3/12) vs. 33.3% (4/12), *p* > 0.05). 

### 2.2. Hemodynamics, Biochemistry Parameters and Blood Gas Analysis

Table 1 displays body weight, hemodynamic change, liver and renal biochemistry parameters of control (*n* = 9) and caffeine-treated (*n* = 8) CBDL rats. In our previous report [17], CBDL rats had significantly higher portal pressure, elevated total bilirubin (TB), aspartate aminotransferase (AST), alanine aminotransferase (ALT), decreased partial pressure of oxygen (PaO_2_) and increased alveolar-arterial oxygen gradient (AaPO_2_) compared to the sham-operated rats, indicating the typical presentation of liver cirrhosis and HPS (see Supplementary Table S1). In the present study, body weight and heart rate were not significantly different between the caffeine-treated and control CBDL rats. Caffeine significantly decreased portal pressure (control vs. caffeine: 17.0 ± 8.1 vs. 10.0 ± 3.7 mmHg, *p* < 0.05). The plasma levels of creatinine, TB, AST, ALT were not significantly influenced by caffeine. The PaO_2_, partial pressure of carbon dioxide (PaCO_2_) and AaPO_2_ in the arterial blood gas analysis were not significantly different either. 

### 2.3. Histopathological Change and Immunochemical Staining of Liver

**Figure 1:**
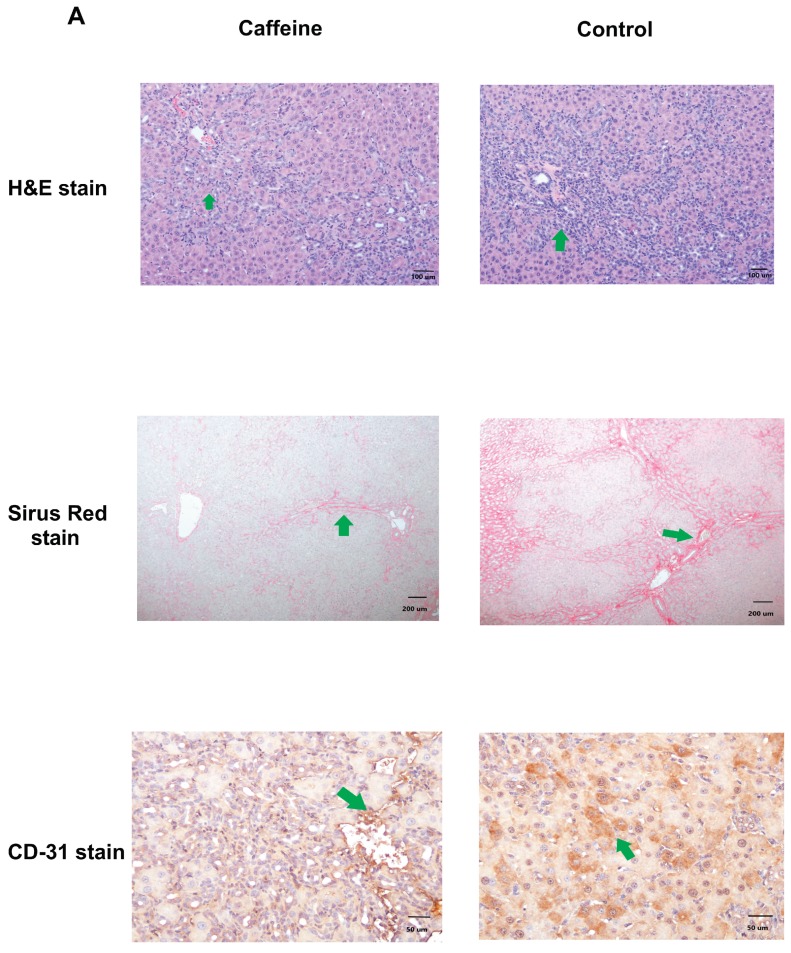

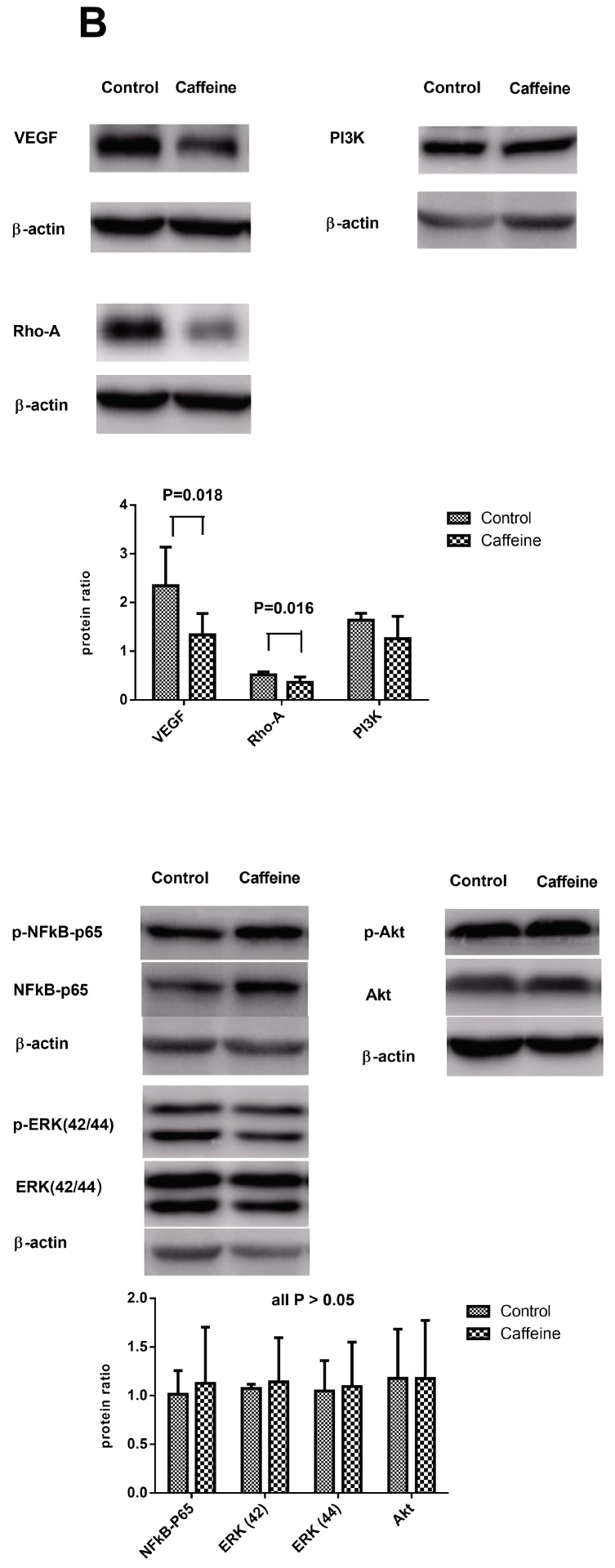
(**A**) Liver histology and immunochemical staining of common bile duct ligation (CBDL) rats treated by caffeine or distilled water (**control**). The representative hematoxylin and eosin (H&E) staining image of control CBDL rats shows ballooning change of hepatocytes accompanied by many inflammatory cells (**green arrow**), indicating the inflammatory change of liver (magnification 100x, upper panel). Liver fibrosis is demonstrated by Sirius red staining (magnification 40x, **green arrow** indicating red area, **middle panel**). As compared with the control group, caffeine significantly attenuates liver fibrosis (**middle panel**). In addition, many CD31-positive staining cells (**green arrow** indicating brown cells) are noted in the control group, which is attenuated by caffeine (magnification 200x, **lower panel**). (**B**) Hepatic protein expressions of caffeine-treated and control CBDL rats. The densitometric quantification and representative Western blots of VEGF and Rho-A kinase, but not PI_3_K, protein expressions are significantly down-regulated by caffeine treatment (VEGF, *p* = 0.018, Rho-A kinase, *p* = 0.016, **upper panel**). The phosphorylated-NF-κB p65, phosphorylated-ERK (42/44), and phosphorylated-Akt protein expressions are not significantly influenced by caffeine (all *p* > 0.05; lower panel). The representative Western blots are shown.

The hepatic hematoxylin and eosin (H&E) staining of CBDL rats showed mononuclear cells infiltration, ballooning change of hepatocytes and destruction of the lobular structure, indicating the inflammatory change of the livers. Sirius red staining revealed the obvious fibrosis of the livers (stained in red), which was significantly attenuated by caffeine. The livers of the control CBDL rats had many CD31-positive staining cells (brown color), which was also attenuated by caffeine (Figure 1A). 

### 2.4. Hepatic Protein Expressions

Figure 1B reveals hepatic protein expressions of CBDL rats treated by vehicle (*n* = 5) or caffeine (*n* = 7). VEGF and Rho-A kinase expressions were significantly attenuated by caffeine treatment (control vs. caffeine: VEGF/β-actin = 2.34 ± 0.79 vs. 1.34 ± 0.44, *p* = 0.018; Rho-A/β-actin = 0.52 ± 0.06 vs. 0.37 ± 0.11, *p* = 0.016; Figure 1B). The phosphoinositide 3-kinases (PI_3_K), phosphorylated- NF-κB p65, phosphorylated-extracellular signal-regulated kinase (ERK) 42/44, and phosphorylated-Akt protein expressions were not significantly influenced by caffeine (PI_3_K/β-actin = 1.64 ± 0.14 vs. 1.26 ± 0.46, phosphorylated-NF-κB p65/NF-κB p65 = 1.01 ± 0.24 vs. 1.13 ± 0.58, phosphorylated-ERK(42)/ERK(42) = 1.07 ± 0.04 vs. 1.14 ± 0.45, phosphorylated-ERK(44)/ERK(44) = 1.05 ± 0.31 vs. 1.09 ± 0.46, phosphorylated-Akt/Akt = 1.18 ± 0.51 vs. 1.18 ± 0.60; all *p* > 0.05). 

### 2.5. Pulmonary Inflammation and Angiogenesis

**Figure 2:**
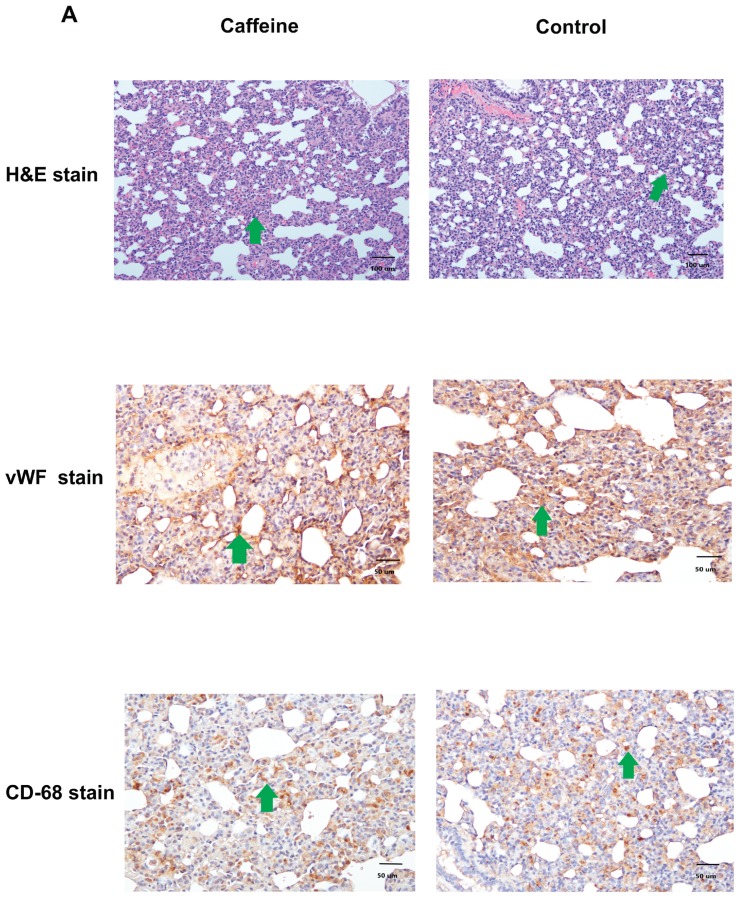

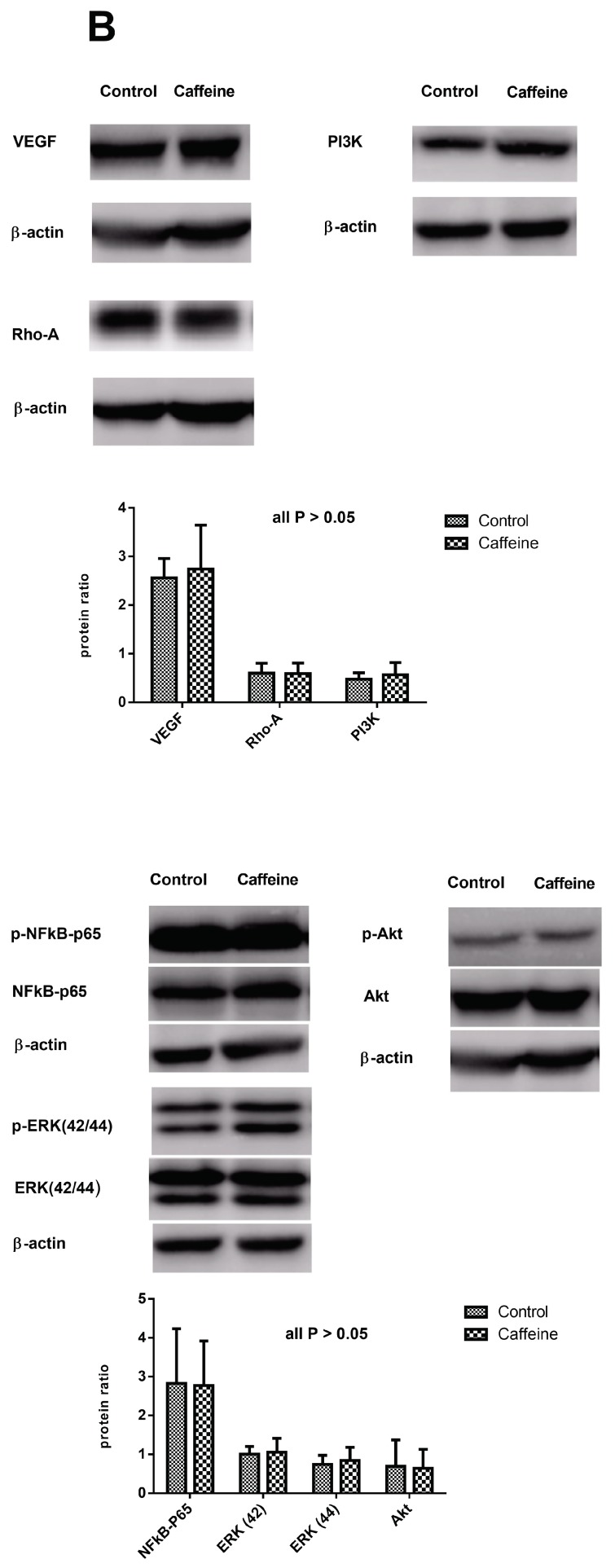

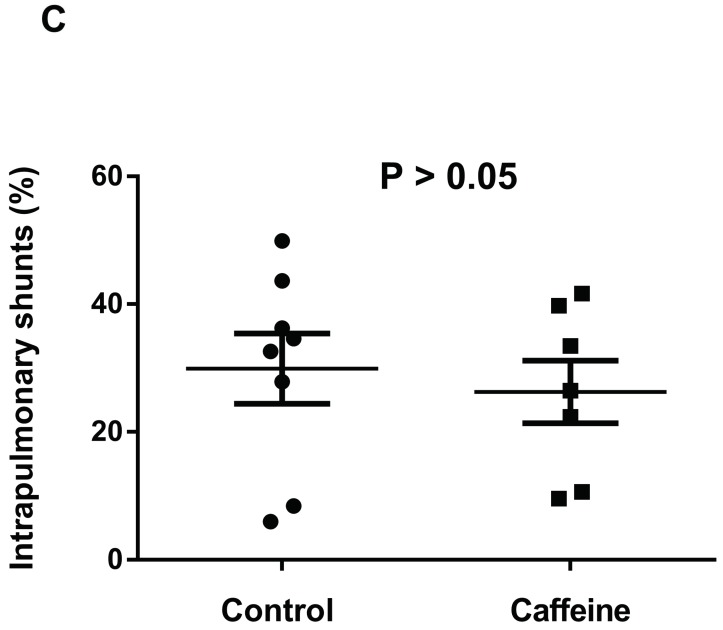
(**A**) Pulmonary histology and immunochemical staining of CBDL rats treated by caffeine or distilled water. The representative H&E staining image depicts prominent polymorphonuclear cells infiltration (**green arrow**) in control and caffeine-treated rats (magnification 100x, upper panel). Caffeine does not diminish vWF-positive staining cells (**green arrow** indicating brown cell; magnification 200x, **middle panel**) as compared to the control rats. Also, the CD68-positive staining cells are not attenuated by caffeine (**green arrow** indicating brown cell; magnification 200x, **lower panel**). (**B**) Pulmonary protein expressions of caffeine-treated and control CBDL rats. The densitometric quantification and representative Western blots of VEGF, Rho-A kinase and PI_3_K are similar between caffeine-treated and control groups (**upper panel**). The phosphorylated-NF-κB p65, phosphorylated-ERK(42/44), and phosphorylated-Akt protein expressions are not significantly influenced by caffeine (**lower panel**). The representative Western blots are shown. (**C**) Degrees of pulmonary shunting in caffeine-treated and control CBDL rats. The shunting degrees are similar between the two groups (*p* > 0.05).

There were many polymorphonuclear cells in the lungs, accompanied by alveolar wall thickening in both caffeine- and vehicle-treated CBDL rats. The CD68-positive staining macrophage (stained in brown) infiltration was not significantly attenuated by caffeine. The numbers of von Willebrand factor (vWF)-staining cells (stained in brown) were not significantly different between control and caffeine-treated CBDL rats (Figure 2A). 

### 2.6. Protein Expressions in the Lungs of CBDL Rats Treated by Vehicle (n = 5) or Caffeine (n = 7)

Figure 2B shows the pulmonary protein expressions of CBDL rats treated by vehicle or caffeine. The VEGF, Rho-A kinase, PI_3_K, phosphorylated-NF-κB p65, phosphorylated-ERK(42/44), and phosphorylated-Akt protein expressions were not significantly different between caffeine-treated and control rats (VEGF/β-actin = 2.56 ± 0.39 vs. 2.74 ± 0.90, Rho-A/β-actin = 0.60 ± 0.20 vs. 0.59 ± 0.22, PI_3_K/β-actin = 0.50 ± 0.13 vs. 0.57 ± 0.25, phosphorylated-NF-κB p65/NF-κB p65 = 2.83 ± 1.40 vs. 2.77 ± 1.14, phosphorylated-ERK(42)/ERK(42) = 1.01 ± 0.20 vs. 1.06 ± 0.36, phosphorylated-ERK(44)/ERK(44) = 0.74 ± 0.23 vs. 0.84 ± 0.34, phosphorylated-Akt/Akt = 0.69 ± 0.68 vs. 0.64 ± 0.49, all *p* > 0.05). 

### 2.7. Intrapulmonary Shunting

Figure 2C reveals intrapulmonary shunting of CBDL rats treated by vehicle (*n* = 8) or caffeine (*n* = 7). The shunting was not significantly different between these two groups (control vs. caffeine: 29.9 ± 15.6 vs. 26.3 ± 13.0 %; *p* > 0.05). 

## 3. Discussion

We had previously reported that both prophylactic caffeine treatment (started from the first day of CBDL) and therapeutic caffeine treatment (started from the 14th day post-CBDL) ameliorated liver fibrosis and portal hypertension [6]. Consistent with those findings, the current study also found that therapeutic caffeine treatment with the same dose for two weeks ameliorated liver fibrosis, alleviated intrahepatic angiogenesis and reduced portal pressure in rats with CBDL-induced cirrhosis. The mean arterial pressure was not significantly altered by caffeine, implicating that caffeine did not exert adverse effects on systemic hemodynamics. Although caffeine ameliorated liver injury as evidenced by decreased ALT, AST and TB levels in rats with thioacetamide- or carbon tetrachloride-induced liver damage [18,19], our present and previous studies [6] show that the plasma levels of ALT, AST and TB were not influenced by caffeine in CBDL rats. This might be ascribed to the severe and relatively non-modifiable bile stasis and inflammation induced by common bile duct ligation and section in CBDL model. Nevertheless, caffeine still ameliorated the liver fibrosis and portal hypertension in CBDL rats with biliary cirrhosis. The anti-inflammatory impact of caffeine on various types of liver damage, indeed, awaits further investigation. On the other hand, the intrapulmonary inflammation and angiogenesis were not reversed by caffeine. The hypoxia and intrapulmonary shunting were not ameliorated either. These findings suggest that caffeine, although capable of alleviating portal hypertension and liver fibrosis, is not able to mitigate HPS in CBDL rats. 

Caffeine is an adenosine A_1_ and A_2A_ receptor antagonist [20]. Adenosine is a potent endogenous regulator of inflammation and tissue repair. Chan et al. showed that caffeine reversed hepatic fibrosis through inhibition of adenosine receptors in cirrhotic mice [21]. In the present study, we found that caffeine significantly ameliorated liver fibrosis and reduced portal pressure in cirrhotic rats, supporting the benefit of using caffeine treatment in cirrhotic patients. 

Emerging evidence shows that the elevated adenosine level observed in ischemic tissue contributes to hypoxia-induced angiogenesis [22]. Extracellular adenosine activates A_2A_ receptors, which stimulates the release of VEGF from the parenchymal cells, followed by endothelial cells proliferation and migration [23]. Through antagonizing the adenosine receptors, caffeine attenuates angiogenesis. In the current study, we demonstrated that caffeine reduced the extent of hepatic CD31-positive staining cells, indicating the attenuation of intrahepatic angiogenesis. Furthermore, caffeine attenuated the hepatic VEGF/Rho-A kinase protein expressions, which are important mediators modulating angiogenesis. Accordingly, our findings suggest that caffeine ameliorates abnormal intrahepatic angiogenesis through, at least in part, inhibition of VEGF/Rho-A kinase pathway. Nevertheless, caffeine did not reduce intrapulmonary vWF-positive staining cells and pulmonary VEGF protein expression. In addition, the intrapulmonary shunts were not influenced by caffeine. According to the discrepant results from lungs and livers, our data indicate that caffeine-related anti-angiogenesis effect is organ-specific in cirrhosis.

In addition to anti-angiogenesis effects, caffeine has been reported to exert beneficial effects on pulmonary inflammation as well as airway remodeling [24]. In a large US cohort study, the inverse association of coffee consumption was observed for deaths from heart disease, chronic respiratory diseases, diabetes, pneumonia and influenza [25]. The researchers postulated that coffee may reduce mortality risk by affecting inflammation, lung function, insulin sensitivity, and depression. The anti-inflammatory effect of caffeine varies with treatment protocols and doses [26]. Li et al. found that chronic caffeine treatment for two weeks or acute high doses (50 mg/kg, 30 min before acute lung injury) significantly attenuated lung edema, hemorrhage, neutrophil recruitment and the inflammatory cytokine expression in mice with acute lung injury. In contrast, acute caffeine treatment at relatively low doses (5, 15, 30 mg/kg) enhanced pulmonary inflammation and damage [26]. However, the present study applying high doses of caffeine (50 mg/kg) to cirrhotic rats showed that the pulmonary polymorphonuclear cells and CD68-positive staining macrophages were not attenuated, indicating a lack of therapeutic effect on pulmonary inflammation in cirrhosis. Since CD68-positive staining macrophages participate actively in initiating pulmonary inflammation and developing HPS [16,17,27], this might be a crucial factor responsible for the failure of caffeine to treat HPS. 

The coffee or caffeine consumption has many other effects on the liver and lung. We summarize these emerging findings in the Table 2 [6,28,29,30,31,32,33,34,35,36,37,38,39,40,41] and Table 3 [24,26,42,43,44,45,46,47,48,49,50]. 

In conclusion, caffeine ameliorated liver fibrosis and reduced portal pressure without adversely affecting systemic hemodynamics in cirrhotic rats. However, caffeine did not ameliorate HPS. Further study of longer treatment durations, prophylactic caffeine treatment applied at the beginning of liver injury or co-treatment with other anti-inflammatory drugs, such as rosuvastatin or TNF-α blockade agents, might be useful to address this issue. 

## 4. Materials and Methods

### 4.1. Animal Model

Male Sprague-Dawley rats weighing 240–270 g at the time of surgery were used. Rats were housed in plastic cages and allowed free access to food and water. Common bile duct ligation (CBDL) was applied as an animal model of liver cirrhosis with HPS according to previous reports [10,11,14]. The rats were fasted for 12 h before the operation. Under Zoletil 50 (Tiletamine + Zolezepam) anesthesia (0.8 mg/kg, intramuscularly), CBDL was performed. A high yield of secondary biliary cirrhosis was noted four weeks after the ligation [51]. To avoid coagulation defects, CBDL rats received weekly vitamin K injections (50 μg/kg intramuscularly). In all experiments, the principles of laboratory animal care (NIH publication no. 86–23, revised 1985) were followed. This study was approved by the Taipei Veterans General Hospital Animal Committee (IACUC 2017–060) (21/6/2017).

### 4.2. Experimental Design

In humans with moderate to high caffeine intake, doses approximate 10–20 mg/kg/day [52]. Based on a report from Ohta et al. that the amount of caffeine in a single cup of coffee (100 mg) is estimated to be equivalent to a 5 mg/kg dose in rodents, 5 and 15 mg/kg can be considered low-dose caffeine treatment, while 50 mg/kg caffeine administration is a high-dose caffeine treatment in mouse model [41]. In the present study, we used caffeine 50 mg/kg/ day as a high-dose caffeine treatment to investigate the beneficial effect of cirrhotic rats. CBDL rats were fed via oral gavage with 50 mg/kg/day caffeine or vehicle (distilled water 5 mL/day, control group) from the 15th to 28th day after CBDL. On the 28th day after CBDL, the body weight, mortality rate, and hemodynamic data were measured. Arterial blood was collected for gas analysis. Venous blood was collected for determination of TB, AST, ALT, and creatinine. Furthermore, lung and liver were dissected for histological examination and immunohistochemical staining to evaluate liver fibrosis, intrahepatic and intrapulmonary angiogenesis and inflammation. Protein expressions were analyzed with Western blotting. In another series with parallel groups, the intrapulmonary shunting was determined with the color microsphere method. 

### 4.3. Systemic and Portal Hemodynamic Measurements

The right internal carotid artery was cannulated with a PE-50 catheter that was connected to a Spectramed DTX transducer (Spectramed Inc., Oxnard, CA, USA). Continuous recordings of mean arterial pressure were performed on a multi-channel recorder (model RS 3400, Gould Inc., Cupertino, CA, USA). The external zero reference was placed at the level of the mid-portion of the rat. The abdomen was then opened with a midline incision, and the superior mesenteric vein was cannulated with a PE-50 catheter connected to a Spectramed DTX transducer. The abdominal cavity was closed, and the PP was recorded on a Gould model RS 3400 recorder.

### 4.4. Biochemistry and Blood Gas Analysis

The femoral artery and vein were cannulated with PE-50 catheters, one day before the experiments. Both catheters were fixed over the back and flushed with a heparin-contained solution. Blood was withdrawn from the femoral vein to determine plasma concentrations of ALT, AST, TB, and creatinine. Arterial blood was withdrawn from the femoral artery for blood gas analysis. Arterial gas exchange was evaluated by the alveolar-arterial oxygen gradient, which was calculated as 150-(PaCO_2_/0.8)-PaO_2_. 

### 4.5. Histopathological and Immunochemical Staining

Liver and lung were dissected free and fixed in 10% neutral buffered formalin. The sections were stained with H&E and examined by light microscopy. The liver sections were stained with Sirius red for 8 min according to the manufacturer’s instructions (Polysciences Inc., Warrington, PA, USA) to determine the extent of collagen deposition [53]. The immunochemical staining was performed with anti-CD68 antibody (diluted 1:200, ab31630, Abcam Cambridge, UK) to detect pulmonary CD68-positive macrophages, indicating the severity of intrapulmonary inflammation. To determine the intrahepatic angiogenesis, immunochemical staining with anti-CD31 antibody (1:200, Serotec and Pharmingen, San Diego, CA, USA) was performed [6]. Anti-vWF antibody (1:100, MCA127T, AbD Serotec, UK) was applied to survey the intrapulmonary angiogenesis [54]. The immunochemical staining was followed by the biotinylated anti-mouse IgG (H+L) (Vector Laboratories, Burlingame, CA, USA) as the second antibody. Detection of biotinylated antibody was performed using the VECTASTAIN^®^-Elite ABC kit from Vector Laboratories. For the chromogen, DAB (3′-diaminobenzidine tetrahydrochloride) was used, which resulted in a brown color at the antigen site.

### 4.6. Western Blot Analysis for Protein Expressions

Lung and liver were immediately frozen in liquid nitrogen and stored at −80 °C until required. Protein was extracted by pulverization in grinder with liquid nitrogen, followed by 1 mL of lysis buffer (phosphate-buffered solution containing 1% Nonidet P-40, 0.5% sodium deoxycholate, 0.1% sodium dodecyl sulfate (SDS)) and 0.05% protease inhibitor cocktail solution (Roche Diagnostics GmbH, Penzberg, Germany) for each 100 mg powdered sample. Protein concentration was determined for each sample by the Bradford method [55]. An aliquot of 20–40 µg protein from each sample that dissolved in sample buffer (63 mmol/l of Tris-HCL, pH 6.8, containing 2% SDS, 10% glycerol, 5% 2-mercaptoethanol, and 0.005% bomphenol blue) and 10 µg positive control were separated on denaturing SDS-10% polyacrylamide gels by electrophoresis (Mini-PROTEAN^®^ 3 Cell, Bio-Rad Laboratories, Hercules, CA, USA). Pre-stained proteins markers (SDS-PAGE Standards, Bio-Rad) were used for molecular weight determination. Proteins were then transferred to a polyvinylidene difluoride membrane (Immum-Blot^TM^ PVDF Membrane, Bio-Rad) by a semi-dry electroblotting system (Trans-Blot^®^ SD Semi-dry Electrophoretic Transfer Cell, Bio-Rad) for 1.5 h at 4 °C. To block non-specific binding, membranes were blocked for 30 min with 3% non-fat dry milk in TBS-T, pH 7.4 (25 mmol/l Tris base-137 mmol/l NaCl-2.7 mmol/l KCL-1% Tween 20). Blots were incubated with the primary antibody [anti-PI_3_K (1:1000; Cell Signaling Technology), anti-NF-κB p65 (1:200; Santa Cruz Biotechnology, Santa Cruz, CA, USA), anti-phosphorylated-NF-κB p65 (1:1000; Abcam plc, Cambridge, UK), anti-VEGF (1:1000; Santa Cruz Biotechnology), anti-RhoA (1:1000; Cell Signaling Technology), anti-Akt (1:500, Cell Signaling Technology), anti-phosphorylated Akt (1:2000, Cell Signaling Technology), anti-ERK, -phosphorylated ERK (1:3000, Millipore Corporation), diluted with 3% non-fat dry milk in TBS-T] for 90 min at room temperature and washed. Then the blots were incubated for 90 min with the secondary antibody (horseradish peroxidase-conjugated goat anti-mouse IgG antibody, diluted with 3% non-fat dry milk in TBS-T, Sigma Chemical Co., St. Louis, MO, USA) and washed. The specific proteins were detected by enhanced chemiluminescence (Immobilon Western Chemiluminescent HRP Substrate, Merk Millipore Co., Billerica, MA, USA). With a computer-assisted video densitometer and digitalized system (BioSpectrum ^®^ 600 Imaging System, Ultra-Violet Products Ltd., Upland, CA, USA), the blots were scanned and photographed, after which the signal intensity (integral volume) of the appropriate band was analyzed. 

### 4.7. Intrapulmonary Shunting Analysis

The degree of intrapulmonary shunting was determined using the color microsphere method [14]. Before microsphere injection, indwelling PE-50 femoral arterial and venous catheters were placed. On the day of measurement, 2.5 × 10^6^ custom mixed and counted cross-linked polystyrene-divinylbenzene microspheres labeled red (size range 6.5–10 μm; Interactive Medical Technologies, Los Angeles, CA) in 0.20 mL of sterile PBS were injected over 2–4 s through the femoral vein catheter, which was immediately flushed with 0.2 mL of sterile PBS over 2–4 s. A reference blood sample was withdrawn from the femoral arterial catheter started at the time of femoral vein injection for a total of 90 s at a constant rate of 1.0 mL/min. The volume removed was replaced with an equal volume of sterile PBS. Samples of beads before venous injection and reference blood samples were coded. The numbers of colored microspheres in the blood preparations were determined using a hemacytometer counting slide having a known volume. Because the diameter of normal pulmonary microvasculature was less than 5μm, most part of microsphere beads injected from femoral vein would be trapped in the lung. However, microspheres would pass through the intrapulmonary shunting vessels in the HPS animal and were collected in the reference blood from the femoral artery. Total numbers of microspheres passing through the pulmonary microcirculation were calculated as reference blood sample microspheres per mL times estimated blood volume. Estimated blood volume (mL) was calculated as: 0.06 x body wt (g) + 0.77 [56]. Intrapulmonary shunting (%) was calculated as: (total number of microspheres passing through the pulmonary microcirculation/total beads injected into the venous circulation) x 100. 

### 4.8. Drugs

Caffeine was purchased from Sigma Chemical Co. (St. Louis, MO, USA).

### 4.9. Data Analysis

The results are expressed as mean ± standard deviation. Statistical analyses were performed with Student’s t test. The survival rate was analyzed with log-rank test. Results were considered statistically significant at a *p*-value less than 0.05. 

## Figures and Tables

**Figure 3:**
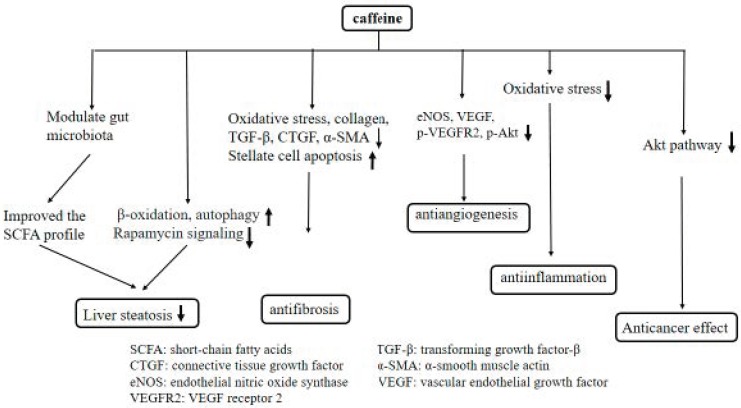
The postulated mechanisms regarding the effect of caffeine on the liver.

**Figure 4:**
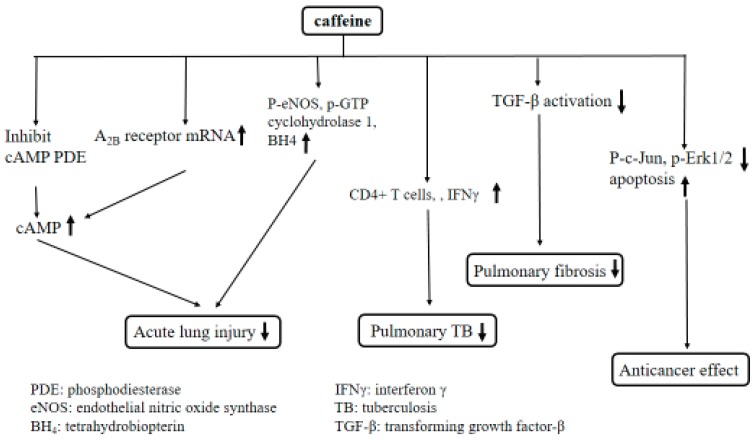
The postulated mechanisms regarding the effect of caffeine on the lung.

**Table 1:** Body weight, hemodynamics, biochemistry and arterial blood gas data in cirrhotic rats treated with or without caffeine.

	Control (*n* = 9)	Caffeine (*n* = 8)
BW (g)	357 ± 54	330 ± 35
MAP (mmHg)	134 ± 17	133 ± 27
PP (mmHg)	17.0 ± 8.1	10.0 ± 3.7 *
AST (IU/L)	625 ± 114	721 ± 78
ALT (IU/L)	216 ± 142	183 ± 55
TB (mg/dL)	7.9 ± 2.3	7.7 ± 0.5
Creatinine (mg/dL)	0.5 ± 0.2	0.4 ± 0.1
PaO_2_ (mmHg)	75.1 ± 10.2	78.6 ± 6.5
PaCO_2_ (mmHg)	37.4 ± 5.3	39.8 ± 5.7
AaPO_2_ (mmHg)	28.1 ± 7.0	21.7 ± 9.1

BW: body weight; MAP: mean arterial pressure; PP: portal pressure; AST: aspartate aminotransferase; ALT: alanine aminotransferase; TB: total bilirubin, Cr: creatinine; PaO_2_: partial pressure of oxygen; PaCO_2_: partial pressure of carbon dioxide; AaPO_2_: alveolar-arterial oxygen gradient; * *p* < 0.05 compared to control group.

**Table 2:** Emerging evidence of coffee or caffeine treatment on various liver diseases.

References	Experimental Model	Major Findings
Wiltberger et al. [28]	Human	Coffee consumption was associated with a decreased risk of hepatocellular carcinoma recurrence and increased survival following orthotopic liver transplantation.
Nishitsuji et al. [29]	Mice	Caffeine would affect the gut dysbiosis and the disrupted plasma short-chain fatty acid profile, then subsequently prevented nonalcoholic steatohepatitis.
Veronese et al. [30]	Human	The consumption of coffee was not associated with liver steatosis either nonalcoholic fatty liver disease or alcoholic fatty liver disease.
Kennedy et al. [31]	Human	Increased consumption of caffeinated and decaffeinated coffee is associated with reduced risk of hepatocellular carcinoma, including in pre-existing liver disease.
Arauz et al. [31]	Rats	Coffee prevented liver cirrhosis by attenuating the oxidant process, blocking hepatic stellate cell activation, and down-regulated the profibrotic molecules.
Wijarnpreecha et al. [33]	Human	Coffee consumption showed a decreased risk of advanced liver fibrosis and inflammation among hepatitis C-infected patients.
Hsu et al. [6]	Rats	Caffeine decreased portal pressures, ameliorated hyperdynamic circulation, protosystemic collateral shunting, mesenteric angiogenesis and liver fibrosis in cirrhotic rats.
Lammert et al. [34]	Human	Coffee consumption was lower among patients with primary sclerosing cholangitis, but no primary biliary cholangitis, compared with controls.
Barcelos et al. [35]	Rats	Caffeine modified the hepatic responses associated to exercise-induced oxidative stress of trained rats.
Sinha et al. [36]	Mice	The caffeine’s lipolytic action was through autophage in mammalian liver and it was a potent stimulator of hepatic autophagic flux.
Li et al. [37]	Cells	The caffeine-enhanced autophagic flux in hepatic stellate cell (HSC) was stimulated by endoplasmic reticulum stress, which further weakened HSC viability via the induction of apoptosis.
Ikeda et al. [38]	Human	There was an inverse association between coffee consumption and elevated aminotransferase in men, and it was more evident in those with high alcohol consumption and in those with low body mass index.
Ong et al. [39]	Human	Caffeine intake does not affect liver stiffness in chronic hepatitis B-infected patients.
Kurozawa et al. [40]	Human	An inverse association between coffee consumption and HCC mortality was found in a large cohort study in Japan.
Ohta et al. [41]	Rats	A high dose of caffeine (100 mg/kg) completely blocked both liver damage and proinflammatory cytokine responses through an A2AR-independent mechanism.

**Table 3:** Emerging evidence of coffee or caffeine treatment on various lung diseases.

References	Experimental Model	Major Findings
Amaral et al. [42]	Mice	Caffeine enhanced the frequency and number of parenchymal CD4+ T-cell and stimulated immune function in severe tuberculosis.
Doyle et al. [43]	Human	Caffeine reduced the incidence of brochopulmonary dysplasia in the new-born period and improved pulmonary function at follow-up.
Jing et al. [44]	Rats	Early caffeine treatment could protect immature lungs from hyperoxia-induced lung injury.
Fehrholz et al. [24]	Cells	Glucocorticoid had adverse effects on long-term remodeling by induction of connective tissue growth factor in lung cells, however, co-treatment with caffeine attenuated connective tissue growth factor expression and promoting restoration of lung homeostatsis.
Tatler et al. [45]	Cells	Caffeine owned anti-fibrotic capacities for the epithelial cells and fibroblasts in the lung.
Chou et al. [46]	Rats	Caffeine could mitigate lung inflammation induced by ischemia-reperfusion of the lower limbs.
Guertin et al. [47]	Human	Coffee drinking was positively associated with lung cancer.
Wang et al. [48]	Cells	Caffeine administration increased the cisplatin-induced lung cancer killings and cellular apoptosis.
Li et al. [26]	Mice	Caffeine either enhanced lung damage by antagonizing A2A receptor or exerted protection against lung damage via A2A receptor-independent mechanisms, depending on the timing of exposure and dose of administration.
Lu et al. [49]	Cells	Caffeine and tea polyphenos inhibited the progression of lung adenoma to adenocarcinoma.
Yoder et al. [50]	Baboons	Early caffeine treatments were associated with better lung function in immature baboons.

On the other hand, we drew the postulated mechanisms regarding the effect of caffeine on the liver (Figure 3) and lung (Figure 4).

## Supplementary Materials

Supplementary can be found at https://www.mdpi.com/1422-0067/20/7/1566/s1.
