# Supplementary material for: Effects of Caffeine Treatment on Hepatopulmonary Syndrome in Biliary Cirrhotic Rats
Source: Int J Mol Sci. 2019 Mar 28;20(7):1566. doi: 10.3390/ijms20071566 (PMC6480428; doi:10.3390/ijms20071566)
Supplement: Supplementary file 1 [file ijms-20-01566-s001.zip › Raw data of WB of caffeine in HPS study.pdf]

Liver

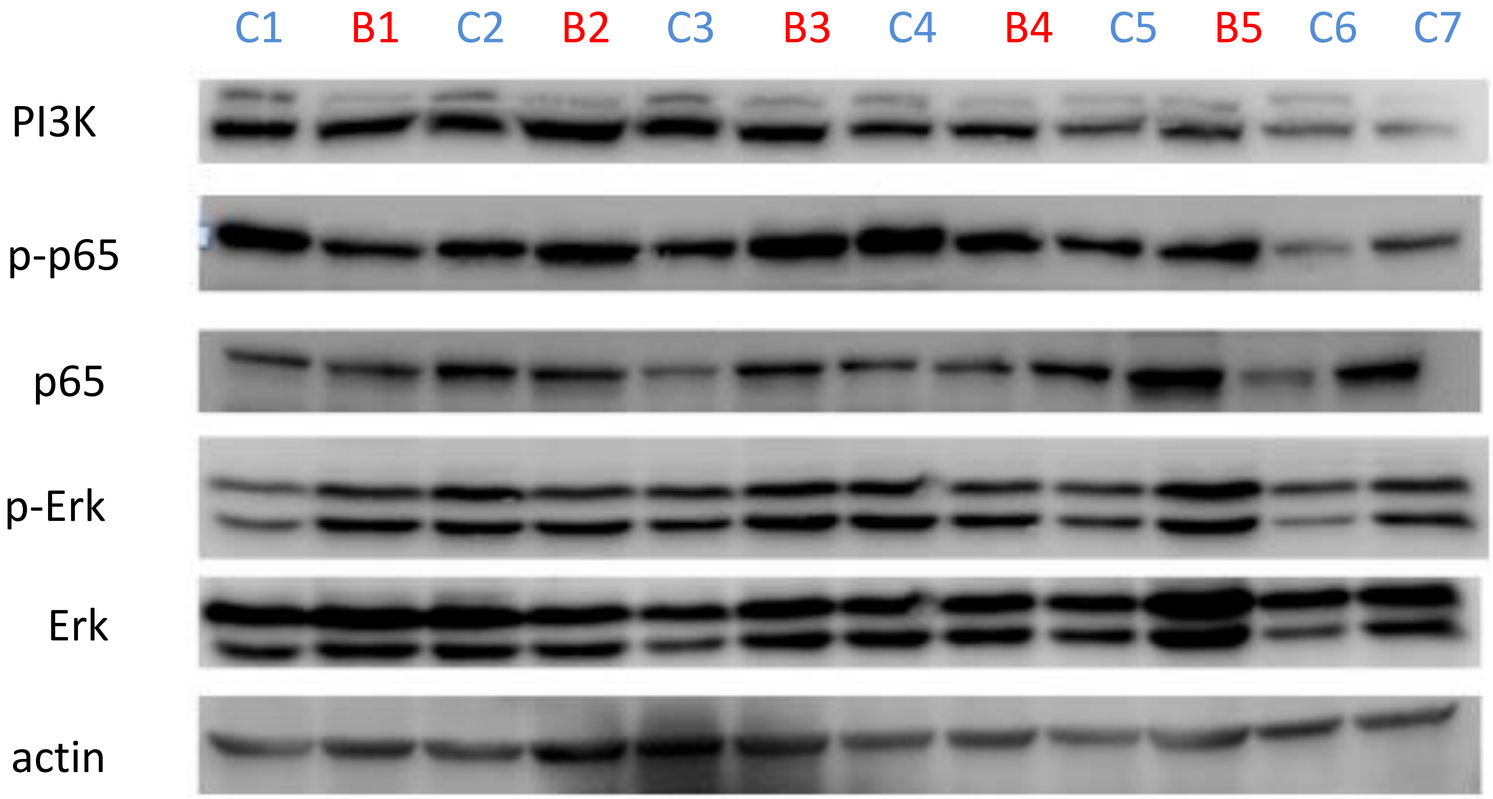

B: BDL (Caffeine 0mg)

C: BDL + Caffeine 50mg

Liver

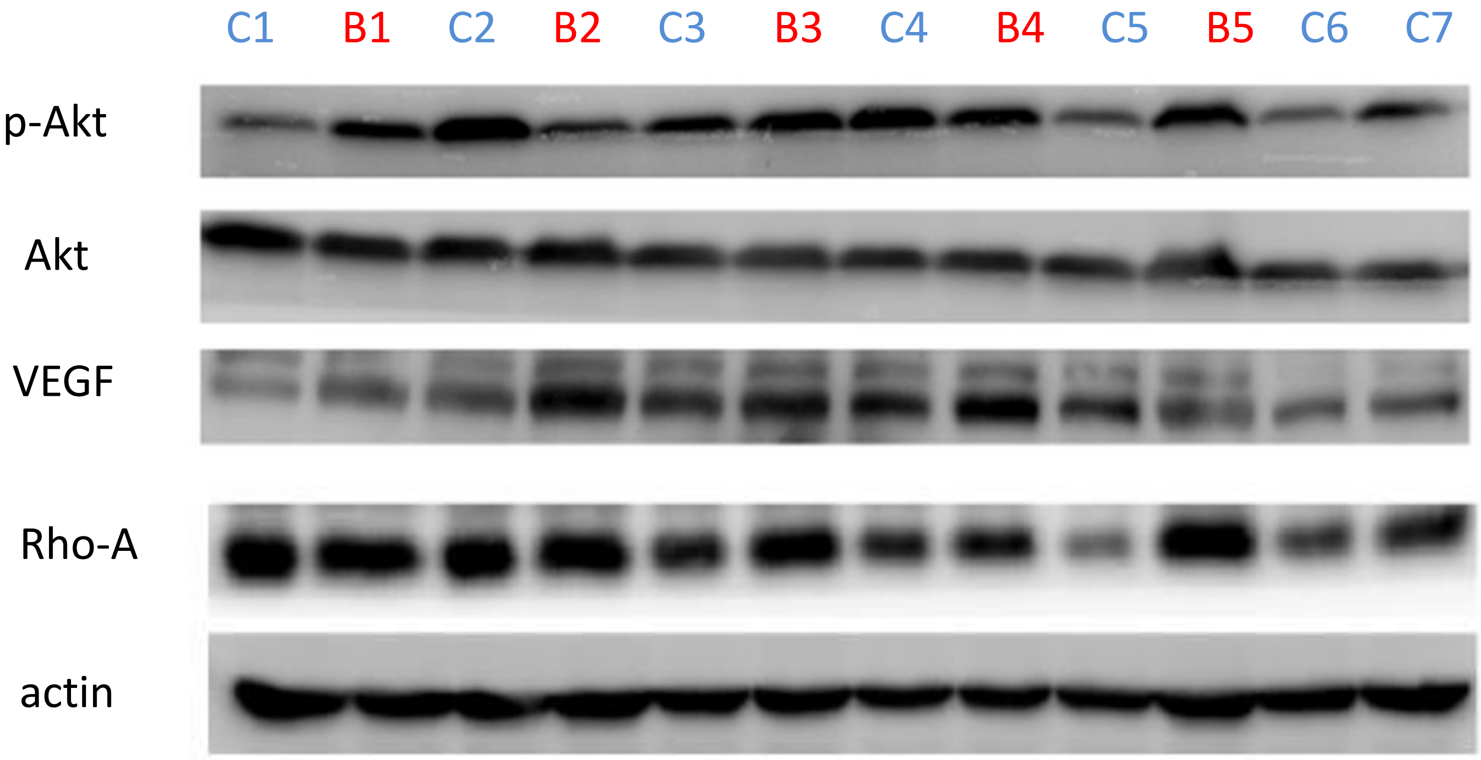

B: BDL (Caffeine 0mg)
C: BDL + Caffeine 50mg

Lung

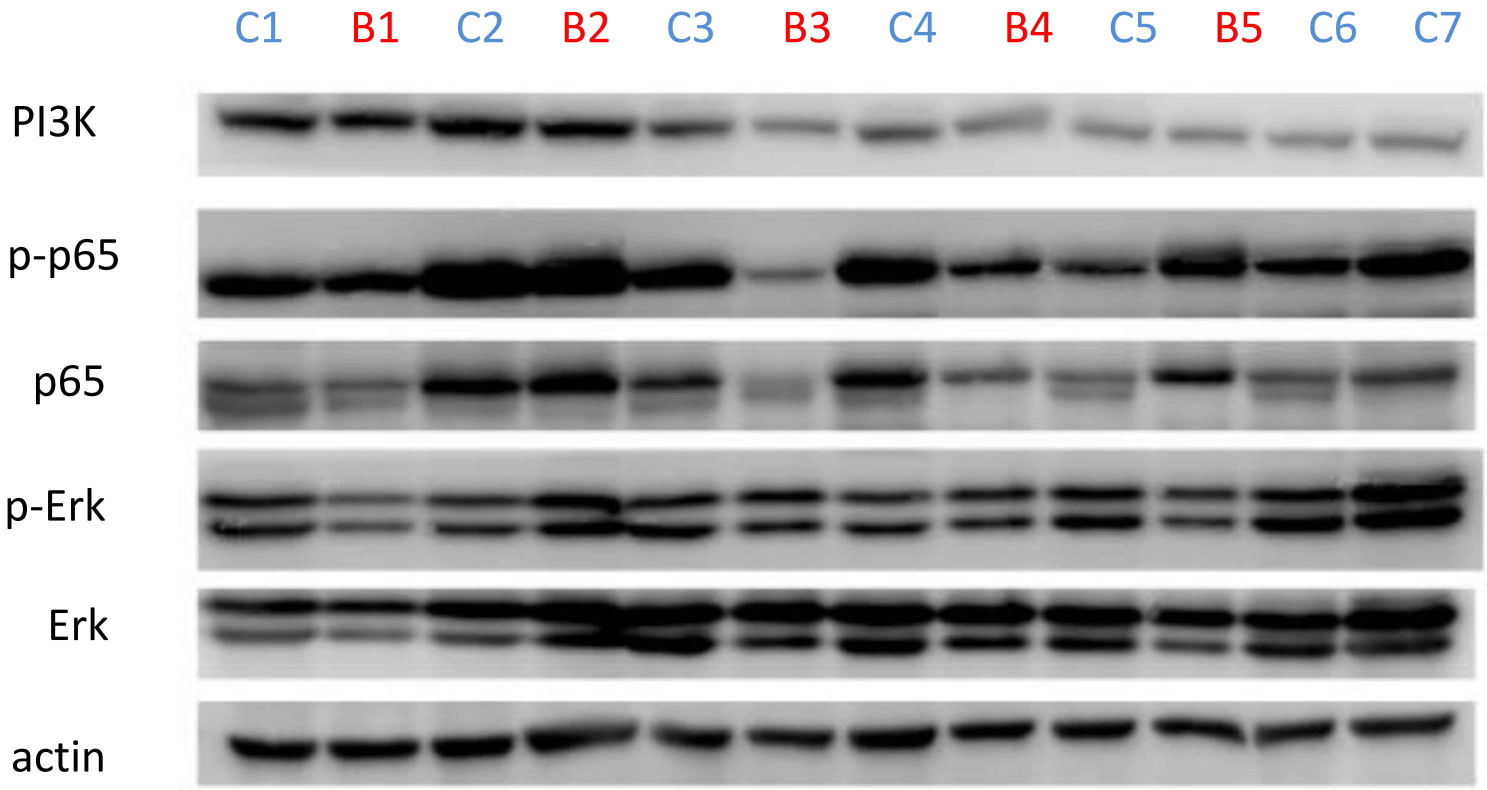

B: BDL (Caffeine 0mg)

C: BDL + Caffeine 50mg

Lung

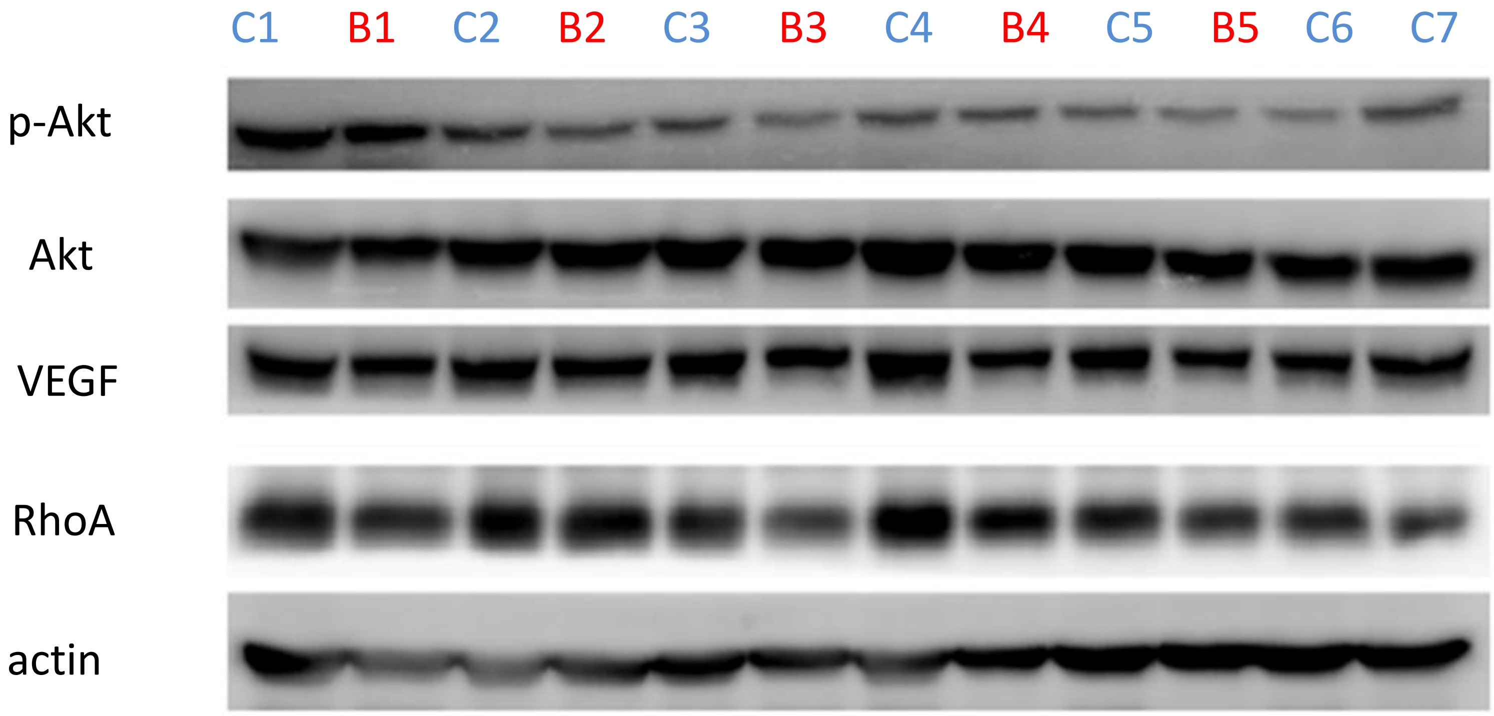

B: BDL (Caffeine 0mg)

C: BDL + Caffeine 50mg
