# Supplementary material for: Effects of Caffeine Treatment on Hepatopulmonary Syndrome in Biliary Cirrhotic Rats
Source: Int J Mol Sci. 2019 Mar 28;20(7):1566. doi: 10.3390/ijms20071566 (PMC6480428; doi:10.3390/ijms20071566)
Supplement: Supplementary file 1 [file ijms-20-01566-s001.zip › Supplementary Table 1.pdf]

Supplementary Table 1. Body weight, hemodynamic parameters, liver biochemistry, and arterial gas analysis of sham and CBDL rats receiving rosuvastatin or vehicle treatment

| | Sham rats | | CBDL rats | |
|---|---|---|---|---|
| | Rosuvastatin (n=11) | Control (n=10) | Rosuvastatin (n=11) | Control (n=10) |
| BW prior (g) | 264 ± 20 | 279 ± 70 | 260 ± 16 | 269 ± 12 |
| BW post (g) | 413 ± 24 | 410 ± 23 | 337 ± 27 [a,]* | 371 ± 27 |
| MAP (mmHg) | 128 ± 13 | 122 ± 14 | 109 ± 14* | 110 ± 14* |
| PP (mmHg) | 6.7 ± 2.3 | 7.5 ± 1.1 | 15.9 ± 2.5* | 15.9 ± 2.4* |
| HR (beats/min) | 398 ± 52 | 394 ± 56 | 353 ± 45 | 369 ± 53 |
| ALT (IU/L) | 74 ± 22 | 76 ± 21 | 201 ± 164* | 234 ± 95* |
| AST (IU/L) | 296 ± 123 | 320 ± 100 | 1362 ± 1162* | 1262 ± 340* |
| TB (mg/dL) | 0.09 ± 0.01 | 0.08 ± 0.01 | 7.96 ± 1.22* | 8.21 ± 1.58* |
| $PaO_2$ (mmHg) | 89.5 ± 4.1 | 91.9 ± 4.9 | 90.7 ± 4.3 [#] | 85.4 ± 5.6 [b] |
| $PaCO_2$ (mmHg) | 39.7 ± 4.0 | 39.1 ± 2.7 | 37.3 ± 3.2 | 36.9 ± 3.5 |
| $AaPO_2$ (mmHg) | 10.9 ± 4.1 | 9.3 ± 2.4 | 12.6 ± 2.2 [a] | 18.5 ± 4.2* |
| VEGF (pg/ml) | NA | NA | 17.0 ± 2.9 [a] | 20.7 ± 4.9 |
| TNF-α(pg/ml) | NA | NA | 20.1 ± 4.4 [a] | 24.9 ± 3.7 |

BW prior: body weight before CBDL or sham operation; BW post: body weight after different treatments; MAP: mean arterial pressure; PP: portal pressure; HR: heart rate; AST: aspartate aminotransferase, ALT: alanine aminotransferase, TB: total bilirubin; $PaO_2$: partial pressure of oxygen; $PaCO_2$: partial pressure of carbon dioxide; $AaPO_2$: alveolar arterial oxygen gradient; VEGF: vascular endothelial growth factor; TNF-α: tumor necrosis factor α; NA: non-analysis; $^{\mathbf{a}}$ $P < 0.05$ compared with CBDL rats with vehicle treatment; $^{b}$ $P < 0.05$ compared with sham rats with vehicle treatment; ***** $P < 0.05$ compared with sham rats with or without rosuvastatin treatment; $^{\#}$ $P = 0.061$ compared with CBDL rats with vehicle treatment.

From Chang, C.C. et al. Rosuvastatin improves hepatopulmonary syndrome through inhibition of inflammatory angiogenesis of lung. Clin. Sci (Lond). **2015**, 129, 449–460.
